# Unmasking the appeal: voices of young adults prior to World No Tobacco Day at Qatar University

**DOI:** 10.3389/fpubh.2026.1837714

**Published:** 2026-05-14

**Authors:** Ohoud Alyafei, Shahd Al-Shafai, Shaikha Al-Mansouri, Rana Abouzoor, Mohammed Al-Hamdani

**Affiliations:** Department of Public Health, College of Health Sciences, QU Health, Qatar University, Doha, Qatar

**Keywords:** flavor, nicotine, Qatar, student-led, tobacco, World No Tobacco Day

## Abstract

**Background:**

The global tobacco industry continues to target young adults through flavored nicotine products and influencer-driven marketing, masking the harmful effects of addiction. The 2025 World No Tobacco Day theme, “Unmasking the Appeal,” calls for exposing these tactics. This study examined perspectives from Qatar University students on tobacco marketing and strategies to promote a tobacco-free campus.

**Methods:**

Secondary data were analyzed from the “Tobacco-Free Generations” event at Qatar University. Participants aged 18–30 provided open-ended responses on raising awareness about flavored tobacco and nicotine addiction. Data were coded using ATLAS.ti (version 23.2.1) and analyzed using thematic analysis. To guide the process, we used the six-step process in Braun and Clarke's framework.

**Results:**

Four overarching themes emerged: health and wellbeing, emphasizing the risks of tobacco and the need for supportive environments; education and awareness, highlighting student-led campaigns, social media engagement, and faculty involvement; policy, regulations, and research, stressing smoke-free campus policies, regulation of flavored products, and integration of tobacco control into curricula; and advocacy and stakeholder collaboration, underscoring partnerships with health authorities and public engagement. Subthemes revealed the importance of tailored strategies to engage youth and counter industry marketing tactics.

**Conclusion:**

Perspectives from students, are critical for shaping effective tobacco control initiatives. Involving these groups in advocacy, education, and policy development can reduce youth susceptibility, counter marketing strategies, and foster a tobacco-free campus, aligning with the goals of World No Tobacco Day 2025.

## Introduction

The tobacco and nicotine industries have adapted their strategies to sustain youth engagement despite global declines in cigarette smoking. These strategies—such as appealing flavors, sleek product designs, and influencer-driven messaging—frame non-combustible products as modern and lower-risk alternatives while obscuring their addictive properties and associated harms (1, 2). The 2025 World No Tobacco Day (WNTD) theme, “Unmasking the appeal: exposing industry tactics on tobacco and nicotine products,” highlights these practices and urges communities to address how flavors and nicotine addiction influence youth across all tobacco and nicotine product categories (2, 3). Products such as e-cigarettes, snus, and nicotine pouches are particularly concerning because they appeal to youth and young adults, re-normalize smoking behaviors, and promote ongoing nicotine uptake (4–7). Many young users also gravitate toward products with high nicotine concentrations (7). Evidence on adult cessation remains inconclusive: while some studies find no flavor-specific advantages, others suggest sustained exclusive e-cigarette use with fruity flavors compared to tobacco flavors, though overall reviews show limited cessation effectiveness (8–10).

Flavored products are a central driver of youth initiation (11, 12), largely by masking the harshness of tobacco (13). Adolescents' perceptions of harm and user identity are shaped by flavor descriptors and promotional strategies (14). Similarly, exposure to e-cigarette advertising increases adolescents' positive attitudes and susceptibility to use (15). Restrictions on flavored product sales have been shown to reduce youth tobacco use, emphasizing the value of comprehensive flavor bans (16). Youth also tend to perceive flavored products as less harmful and more socially acceptable relative to cigarettes, reinforcing patterns of experimentation and continued use (17). Given that most nicotine initiation occurs in youth and early adulthood, removing flavored products from the marketplace remains a key public health priority.

Digital platforms further amplify product appeal. Influencers and peer-generated content normalize flavored tobacco and e-cigarettes, increasing susceptibility among younger audiences (18, 19). Most smokers begin before age 25, and flavored products often reinforce long-term nicotine dependence (20, 21). Evidence shows that flavored e-cigarettes, in particular, strengthen positive perceptions and uptake among youth (22). At the population level, 21.8% of new cigarette use is attributed to previous e-cigarette use (23), and the odds of smoking initiation among never-smokers who use e-cigarettes are 3.19 times higher relative to non-users (24). Smoking relapse is also significantly more likely among current and former e-cigarette users (25). Former e-cigarette users frequently recommend stricter control of flavors and nicotine concentrations, reflecting lived experience with dependence (26).

In the Gulf region, flavored tobacco and nicotine use among youth remains a persistent concern, influenced by cultural acceptance and incomplete enforcement of digital marketing restrictions (27). Qatar's ban on e-cigarettes adds regulatory complexity as international industry tactics increasingly target consumers through online channels (28). Universities, as influential educational environments, are well-positioned to advance awareness and implement policies aligned with WNTD goals. The 2025 theme emphasizes the need for strengthened packaging regulations such as plain packaging— standardizing packages to appear in a single background color, bland text color and size, and no brand image— and large graphic health warnings—to reduce product appeal and counter misleading industry imagery, particularly among young people (2, 29, 30). These measures align with the WHO Framework Convention on Tobacco Control and aim to prevent initiation across both traditional and emerging nicotine products (2, 31). Ongoing updates to packaging and flavor regulations are essential to prevent industry circumvention (32).

WNTD's focus on endgame strategies underscores the importance of regulating flavored products and nicotine concentrations to curb youth uptake of tobacco alternatives, including vaping (2, 33). The WHO notes that flavored nicotine products are a growing contributor to youth nicotine exposure, requiring comprehensive bans and stronger regulatory oversight (2). In Qatar, tobacco use resulted in 401 deaths in 2021 (7.92% of all deaths), with a higher burden among males (9.5%) than females (2.97%), reinforcing tobacco's sustained public health impact (34). To advance a tobacco-free future, endgame policies must incorporate targeted, comprehensive measures across all tobacco and nicotine products, consistent with global WNTD priorities.

### Gap and rationale

Global research highlights the influence of social media and flavored nicotine products on youth initiation; however, evidence from Gulf university settings remains limited. Little is known about how students perceive their responsibility in countering these industry tactics or how universities can translate the WNTD 2025 theme into effective, context-appropriate action. Understanding these perspectives is critical for developing culturally grounded, campus-based interventions that align with national priorities and global tobacco control goals.

### Aim

This study focuses on two key questions:

How do students perceive their role in raising awareness about flavored tobacco and nicotine addiction and its influence on youth?

How can Qatar University take an active role in addressing World No Tobacco Day 2025′s theme, “Unmasking the Appeal,” by highlighting the negative effects of flavors and nicotine addiction?

The study aims to examine how members of the university community view their responsibilities in addressing flavored tobacco use and nicotine dependence. Specifically, it seeks to: (1) assess perceptions of students regarding their role in raising awareness of flavored products, nicotine addiction, and related health risks; and (2) identify ways through which Qatar University can meaningfully support the WNTD 2025 theme by emphasizing the harms associated with flavor additives and nicotine dependence. These insights will inform practical, university-led initiatives that strengthen tobacco control efforts within the local context.

## Materials and methods

### Study design

This study used a qualitative design with open-ended survey responses. We used a phenomenological perspective to convey participants' subjective meanings and perceptions regarding tobacco awareness, prevention, and institutional responsibility within the university setting. While the data did not involve in-depth accounts of lived experience, this phenomenologically informed approach supported interpretation of how participants understood and articulated their roles and expectations in relation to tobacco control, given that their responses were provided following the attendance of a tobacco control event that celebrated WNTD.

### Participants and procedure

Data were derived from the “Tobacco Free Generations” event held at Qatar University, an awareness initiative designed to engage students in discussions about achieving a tobacco-free future. The event featured interactive educational booths aligned with the 2025 WNTD theme, “Unmasking the Appeal,” which focuses on the deceptive attractiveness of flavored tobacco and nicotine products.

The booths were organized and staffed by students from the Department of Public Health, who provided informational materials and engaged attendees in brief discussions related to tobacco use and prevention. During these interactions, students attending the event were invited to voluntarily complete an electronic survey. Participation was open to Qatar University students aged 18 years and older. Individuals under the age of 18 and those not affiliated with Qatar University were excluded. Participants were informed that their responses would be reported anonymously and in aggregated form.

A total of 113 student responses were included in the analysis. The survey consisted of a short demographic section capturing gender, tobacco use status (current user, former user, or never used), and university affiliation (health-related or non-health clusters), followed by two open-ended questions:

Q1) In your opinion, how can students raise awareness about flavored tobacco and nicotine addiction?
Q2) How can Qatar University actively support the World No Tobacco Day theme of “Unmasking the Appeal?”

### Data source and ethical considerations

The data were classified as secondary because the survey responses were originally collected for awareness and engagement purposes during the WNTD event and not for a specific research study. The research team accessed the de-identified responses after the event. Ethical approval for secondary data analysis was obtained from Qatar University's Institutional Review Board (QU-IRB 209/2025-EM).

### Data analysis

Analysis was conducted using ATLAS.ti software (version 23.2.1) (35). Data were analyzed using thematic analysis following Braun and Clarke's six-phase framework (36). All authors except the last author participated in the coding process. Each response was coded by at least two researchers working sequentially. In each pair, the first coder conducted initial coding of the data, followed by the second coder, who reviewed the coded material and added, refined, or reorganized codes as needed. Coding decisions were then discussed among the research team, and disagreements were resolved through consensus. The authors first familiarized themselves with the data through repeated reading, noting participants' strategies, views, and recommendations. Codes were generated inductively, staying close to participants' language. Similar codes were grouped into categories, which informed the development of subthemes and overarching themes. Themes were reviewed in an interactive process to ensure consistency and coherence, and representative quotes were selected to illustrate each theme.

Data adequacy was assessed through ongoing comparison of codes and themes during analysis. No new codes or themes emerged after approximately 100 responses, suggesting thematic saturation. Nevertheless, analysis continued until all completed surveys (*N* = 113) were coded to confirm saturation. This approach was intentionally adopted given the use of brief open-ended survey responses, which typically provide less depth per participant than qualitative interviews and therefore require a larger number of responses to support robust thematic analysis.

### Researcher reflexivity and rigor

Given the researchers' backgrounds in public health, reflexivity was actively incorporated throughout the analysis. Coders engaged in regular discussions to reflect on how their disciplinary perspectives and prior knowledge of tobacco control might shape interpretation. Deliberate efforts were made to distinguish researcher assumptions from participants' expressed views. The use of sequential multiple coding, team-based consensus discussions, and iterative theme refinement contributed to the credibility and transparency of the analysis.

## Results

### Demographic characteristics

A total of 113 students who met the inclusion criteria completed the survey after attending the event. All of them were between 18 and 30 years old. The majority were female (*n* = 90, 79.6%), while 23 (20.4%) were male. In terms of tobacco use, 108 participants (95.6%) reported never using tobacco; two (1.8%) were former users; and three (2.6%) had used tobacco within the past 30 days. Most participants (*n* = 89, 78.8%) were enrolled in health-related colleges such as Health Sciences, Pharmacy, or Medicine, while 24 (21.2%) were from other colleges. Table 1 presents the primary demographic characteristics of the study participants.

**Table 1:** Demographic characteristics of study participants (*N* = 113).

Demographic characteristic	Category	*n*	%
Gender	Female	90	79.6
	Male	23	20.4
Tobacco use status	Never used	108	95.6
	Ex-user	2	1.8
	Used in past 30 days	3	2.6
College cluster	Health-related	89	78.8
	Other	24	21.2

### Themes

The thematic analysis produced four interconnected themes describing participants' perspectives on tobacco control within university and community settings: (1) Health and Wellbeing, (2) Education and Awareness Programs, (3) Policy, Regulations, and Research, and (4) Advocacy and Stakeholder Collaborations. Together, these themes illustrate a shared emphasis on comprehensive, multisectoral approaches to reducing tobacco and nicotine use among youth.

Table 2 presents these primary themes along with their corresponding subthemes, which emerged from participants' responses.

**Table 2:** Themes and subthemes of the qualitative analysis of the participants' responses.

Theme	Subtheme
Health and wellbeing	Chronic conditions worsen by tobacco
	Stress and mental health awareness
	Supportive environment
Education and awareness programs	Launching awareness campaigns
	Leading awareness campaigns
	Social media campaigns by students
	Workshops and community events
Policy, regulations, and research	Tobacco control education in courses and research
	Research and advocacy involvement for policy change
	Regulations for smoking restrictions
	Keeping the momentum against tobacco use
Advocacy and stakeholders' collaborations	Collaborations with stakeholders
	University advocacy led by students

#### Theme 1: health and wellbeing

Participants emphasized the health consequences of tobacco use and the importance of supportive environments that reduce initiation and encourage cessation. Three subthemes emerged.

##### Subtheme 1: chronic conditions worsened by tobacco

Participants recognized tobacco use as a major contributor to chronic disease. They noted the value of clinical expertise and personal testimony in raising awareness, with one explaining that

“*clinicians…explain diseases such as lung cancer and fibrosis…smoking…the main cause of many morbidities.”* (p 31, F, Health Cluster)

Also the importance of raising awareness against tobacco's effect and harm on oral health was emphasized, as one stressed that...

“…*is our role to raise awareness and prevent the use of tobacco, as its effects go unnoticed and can harm both the body and oral health***.”** (p 23, M, Health Cluster)

Participants viewed patient experiences as impactful educational tools:

“*Conferences featuring patients with severe complications…would be very convincing.”* (p 29, F, Health Cluster)

One point emphasized by participants is that short-term disadvantages should be addressed more effectively for young people, as they are less responsive to long-term health risks, as one suggested

“*show the youth about some of the short term drawbacks as the youth in general do not fully realize or are able to comprehend long term plans*.” (p 24, M, Health Cluster)

##### Subtheme 2: stress and mental health awareness

Stress was frequently identified as a driver of tobacco and nicotine use. As one participant expressed,

“*Stress is prevalent nowadays, and so are these products.”* (p 28, F, Health Cluster)

Universities were seen as suitable settings to integrate mental health support with tobacco prevention, given their reach among youth:

“*Having sessions about these issues in university is ideal.”* (p 28, F, Health Cluster).

Participants also emphasized the emotional dimensions of smoking, referring to

“*the feeling people get… the need to smoke, the addiction.”* (p 29, F, Health Cluster)

Supportive communication was considered essential to enhance receptivity to prevention messages:

“*To make smokers feel seen and validate their feelings so they will listen.”* (p 29, F, Health Cluster).

Because stress can contribute to risky or unhealthy behaviors in students, awareness and motivation are needed to support students and reduce their engagement in harmful activities, as one emphasized

“ *students get into… such bad behavior is because of the too much stress that they encounter …so we should raise awareness. We need to motivate students in their studies so they don‘t have to commit any of these illegal recrational activities*.” (p 17, M, Health Cluster)

##### Subtheme 3: supportive environment

Participants highlighted the influence of social environments on smoking-related behaviors. Peer support was seen as crucial:

“*Students could have conversations with their friends who are tobacco users and warn them about its hazardous effects.”* (p 37, F, Health Cluster)

Open discussions and critical engagement were viewed as empowering strategies:

“*They foster a culture of prevention and critical thinking that empowers youth to make healthier choices.”* (p 110, M, Health Cluster)

and another added

“…*sharing accurate information, promote open discussions and support tobacco-free environments. This encourages youth to make healthier choices*.” (p 55, M, Health Cluster)

and another suggested providing resources and counseling for students is one-way universities can support tobacco cessation

“*The university can also provide resources and counseling for students who want to quit using tobacco*.” (p 86, M, Non-Health Cluster)

#### Theme 2: education and awareness programs

Participants described education and awareness initiatives as central to countering tobacco industry tactics and informing youth about health risks.

##### Subtheme 1: launching awareness campaigns

Awareness campaigns were considered vital for communicating the harms of flavored tobacco and nicotine products. Participants emphasized campaigns that

“*highlight the harmful effects of flavored tobacco products and their role in encouraging nicotine addiction, especially among youth.”* (p 15, F, Health Cluster)

and another highlighted that

“*Students and faculty play a crucial role in raising awareness about flavored tobacco and nicotine addiction by organizing educational campaigns and peer-led discussions*..” (p 14, M, Health Cluster)

and

“*implementing comprehensive educational programs that highlight the negative effects of flavored tobacco and nicotine addiction*.” (p 69, M, Health Cluster)

Youth-centered strategies—personal narratives, and collaboration with student clubs—were viewed as particularly effective

“*Qatar University can help…working with student clubs to share real stories and facts.”* (p 66, F, Health Cluster)

##### Subtheme 2: leading education campaigns

Structured educational activities, including lectures, discussions, and seminars, were consistently endorsed. Participants stressed the need to address overlooked issues such as passive smoking:

“*They definitely should address passive smoking, it is as bad as smoking itself.”* (p 29, F, Health Cluster)

They also emphasized combined faculty–student involvement:

“*Students and faculty can make a big impact by educating others about the dangers of flavored tobacco and nicotine addiction.”* (p 48, F, Health Cluster)

Printed and digital materials were seen as valuable reinforcement mechanisms:

“*Students can also help raise awareness by distributing pamphlets…sent via email or physical copies.”* (p 113, F, Health Cluster)

Healthier behaviors among youth can be positively influenced by open dialogue and evidence-based information, as one stated

“*By fostering open conversations and promoting evidence-based information, they can influence youth to make healthier choices*.” (p 14, M, Health Cluster)

##### Subtheme 3: social media campaigns by students

Social media was viewed as a powerful channel for youth engagement and rapid information dissemination. Participants highlighted the value of concise, relatable content:

“*Student campaigns and social media can help spread the message and promote tobacco-free choice.”* (p 2, F, Health Cluster)

and one suggested

“*using social media can help spread awareness among youth*.” (p 81, M, Health Cluster)

Creative formats, such as challenges or short videos, were suggested to expand reach:

“*Engaging students in creative projects…can amplify awareness.”* (p 9, F, Health Cluster)

Social media posts were seen as effective tools for highlighting health risks:

“*Social media to show the dangers of nicotine and flavored tobacco.”* (p 102, F, Non-Health Cluster); another stated “*no smoking posts on social media”* (p 100, M, Non-Health Cluster)

##### Subtheme 4: workshops and community events

Workshops and outreach events were described as opportunities to address industry tactics and correct misconceptions. Participants emphasized events that explain

“*how these products are marketed to appeal to youth and the long-term health risks they carry.”* (p 9, F, Health Cluster)

and another highlighted events that spread awareness and discussing health risks of tobacco use

“*Making events, Spreading awareness, Discussing health risks*” (p 89, M, Non-Health Cluster)

Expert involvement was viewed as essential for credibility and impact:

“*The university can collaborate with health experts to…educate students about how these products are designed to attract young users.”* (p 103, F, Non-Health Cluster)

Involving faculty from different disciplines helps eliminate myths about flavored tobacco and promotes a tobacco-free lifestyle on campus as one suggested

“*involving faculty from health sciences, media, and psychology can provide a multidisciplinary approach to debunk the myths around flavored tobacco and promote a tobacco-free lifestyle on campus*.” (p 75, M, Health Cluster)

Testimonies from ex-smokers were consistently described as emotionally compelling and persuasive.

#### Theme 3: policy, regulations, and research

Participants highlighted the importance of institutional and policy-level measures in supporting sustained tobacco control.

##### Subtheme 1: tobacco control education in courses and research

Integrating tobacco control content into curricula was seen as essential for building critical thinking and awareness. One participant explained:

“*Integrating tobacco related topics into classes can…encourage critical thinking about addiction and public health.”* (p 14, F, Health Cluster)

and another stated

“*integrating relevant topics into health and science curricula, they help inform young people about the risks and long-term consequences of nicotine use*.” (p 110, M, Health Cluster).

Evidence-based instruction was emphasized:

“*By faculty incorporating scientific research…arming students with the ability to make informed decisions.”* (p 98, F, Non-Health Cluster)

Participants supported collaborative research on marketing practices and youth-targeted strategies.

##### Subtheme 2: research and advocacy involvement for policy change

Participants emphasized the role of research and advocacy in shaping public health policy. They noted that student involvement contributes to meaningful change:

“*Their involvement in research and advocacy…helps shape policies that reduce access to flavored tobacco.”* (p 1, F, Health Cluster)

Faculty mentorship was viewed as a key mechanism for linking research findings with practice, as one expressed

“*Through research, mentorship, and peer-to-peer advocacy, they also foster a culture of prevention and critical thinking that empowers youth to make healthier choices*.” (p 110, M, Health Cluster).

##### Subtheme 3: regulation for smoking restrictions

Clear and enforced regulations were seen as essential for reducing tobacco use. Awareness alone was considered insufficient:

“*Regulations that restrict smoking would be more helpful.”* (p 29, F, Health Cluster)

Broader measures, such as banning nicotine use, fines and restrictions on youth access, as one recommended

“*Ban the use of nicotine because of how much of danger it has on the human health*.” (p 89, M, Non-Health Cluster).

##### Subtheme 4: keeping the momentum against tobacco use

Participants stressed the importance of continued engagement throughout the academic year. They believed ongoing visibility strengthens prevention efforts:

“*Having more themes like that could be useful…So keep the energy up!”* (p 27, M, Health Cluster).

Sustained dialogue was seen as crucial for normalizing non-smoking behaviors:

“*When we talk about it in schools or universities, it creates a space where youth can actually understand the risks.”* (p 6, F, Health Cluster).

#### Theme 4: advocacy and stakeholder collaborations

Participants emphasized that effective tobacco control requires coordinated contributions from external partners, students, and institutional leadership.

##### Subtheme 1: collaborations with stakeholders

Partnerships with health institutions such as HMC, PHCC, and MOPH were perceived as central to strengthening advocacy, enhancing program credibility, and supporting cessation:

“*Collaborating with local health authorities can further amplify the message.”* (p 11, F, Health Cluster)

and

“*Collaborating with health authorities can help spread awareness among youth*.” (p 81, M, Health Cluster)

and another emphasized

“*The university can collaborate with health organizations to host expert talks, interactive exhibitions, and student-led forums*” (p 77, F, Non-Health Cluster)

Joint social media advocacy was viewed as a means of combating misinformation.

##### Subtheme 2: university advocacy led by students

Students were described as influential advocates capable of leading relatable, engaging initiatives. Participants emphasized student-led discussions, campaigns, and artistic or social media-based efforts:

“*Getting student clubs involved would make it more engaging.”* (p 6, F, Health Cluster).

Collaboration between students and faculty was seen as crucial for reinforcing a unified message:

“*Faculty and students can work together to spread awareness.”* (p 48, F, Health Cluster)

Additionally, allowing healthcare students to engage in school outreaches and developing educational video materials about tobacco use on health implications, as one suggested

“*Qatar university can allow healthcare students to go to schools or make awarness videos in regards its impacts on health*.” (p 23, M, Health Cluster).

##### Subtheme 3: advocacy against flavors and nicotine

Participants supported the university's involvement in advocating against flavored products:

“*Promote and be a part of the movement in banning flavored tobacco flavors.”* (p 3, F, Health Cluster)

Campus-wide smoke-free policies received strong endorsement:

“*Ban smoking on campus grounds.”* (p 19, F, Health Cluster)

Advocating tobacco-free policies and supporting prevention initiatives can diminish the appeal and availability of nicotine products in the community as one expressed

“*By promoting tobacco-free policies and supporting prevention programs, students and faculty work together to reduce the appeal and accessibility of nicotine products in their communities*.” (p 75, M, Health Cluster).

Visible advocacy—through posters, booths, and digital content—was described as essential for maintaining awareness. Department-level initiatives were recommended to ensure consistent messaging across campus.

## Discussion

The participants' responses offered valuable insights into critical factors and strategies for effective tobacco-control initiatives in Qatar. Their perspectives emphasized four interconnected themes: health and wellbeing; education and awareness programs; policy, regulations, and research; and advocacy and stakeholder collaborations. These themes demonstrate how participants conceptualize tobacco use not only as a personal health concern but also as a social, educational, and policy issue that requires coordinated action within and beyond the university. The findings suggest that students view tobacco control as a shared responsibility rather than solely an individual behavior-change issue, highlighting the central role universities can play as normative and regulatory environments. The following sections discuss each theme in relation to existing evidence and explore implications for strengthening tobacco-control efforts at Qatar University and other higher education institutions.

In the first theme, participants underscored that tobacco control is essential for improving health. Three subthemes emerged: tobacco's role in worsening chronic disease and how former users through narratives and experts through their knowledge can improve the issue, the connection between stress and smoking, and the importance of supportive environments. Specifically, participants did not merely acknowledge health risks; they emphasized how health information should be communicated—through experts, lived experience, and short-term consequences—to resonate effectively with young adults. The role of experts as emphasized by participants in our study is echoed by studies finding that smoking cessation, through experts is crucial to prevent the progression of disease in patients with chronic conditions (37, 38). Participants also identified stress and mental health challenges as major drivers of tobacco initiation and continuation, aligning with evidence showing that stress, anxiety, and emotional strain significantly predict smoking behavior (39–41), and nicotine's temporary relief from tension reinforces its use (42), making stress-management support really important (43). This highlights that prevention messaging could benefit from being paired with mental health–informed approaches that acknowledge why young people initiate or continue nicotine use, at least with respect to university settings. Participants further emphasized the value of supportive social and institutional environments. Campus norms, peer influence, and smoke-free policies shape smoking attitudes and behaviors (44–46), and participants in this study highlighted the importance of educational and peer-based strategies to reduce acceptability and promote cessation. This aligns with studies showing that social networks play a critical role in supporting quitting (47). The above-described perceptions of participants in this study and their alignment with past literature suggest that there may be support for integrating stress-management and mental health aspects into tobacco-control initiatives, smoke-free policies and peer-led programs.

The second theme highlighted education and awareness campaigns as central to preventing tobacco and nicotine use. Participants viewed these programs as vital for guiding individuals toward a tobacco-free future and identified four subthemes: awareness campaigns, formal education initiatives, student-driven social media engagement, and community workshops. While these strategies were presented descriptively, their importance lies in how participants emphasized the delivery and credibility of information rather than information alone. These subthemes emphasize collaboration among students, faculty, and public health institutions to enhance tobacco-related knowledge and shape healthier attitudes. Participants noted that targeted awareness programs effectively counter industry messaging, particularly about flavored products. Faculty–student collaboration was identified as a key element for sustaining engagement and promoting inclusive, ongoing dialogue. Participants also emphasized the need to address passive smoking and misconceptions surrounding alternative nicotine products, which are valid concerns given that 46.2% of a large sample of Qataris and long-term Qatari residents report living with at least one smoker 39.1% report daily exposure to second-hand smoke (48). Social media was viewed as especially effective for reaching younger, digitally oriented populations, consistent with research showing that digital platforms allow wide-reaching and cost-effective campaigns (49–52). Participants also stressed the importance of integrating cessation resources into educational efforts. Together, these findings suggest that students support multi-channel education strategies that combine traditional learning, digital engagement, and accessible cessation support to promote long-term behavior change in university settings.

In the third theme, participants emphasized policy action, curricular integration, research engagement, and sustained advocacy as central to addressing tobacco use among young people. Universities were seen as both educational settings and environments that shape social norms. Participants' support for policy measures demonstrates their appreciation that individual behavior change is governed by institutional rules and enforcement, and that education alone is insufficient without structural support. Integrating tobacco control content into coursework was viewed as essential for preparing students to understand addiction, industry marketing practices, and long-term health implications. Evidence supporting health education in university settings reinforces this view (53). Participants also highlighted the importance of research and advocacy, noting that student involvement can influence campus norms and national strategies. This is especially relevant in the Middle East, where culturally informed evidence is needed to shift attitudes and reduce youth susceptibility to tobacco use, especially for products like waterpipe (54). Student-led projects—such as analyzing flavored product marketing or documenting peer smoking norms—were described as potential catalysts for advocacy through social media and campus events. Participants strongly supported smoke-free campus policies, stricter enforcement, and collaboration with health authorities, consistent with evidence showing that policies that are fully implemented and adhered to show far stronger reductions in smoking-related outcomes (55). They also stressed regulating flavored products, given their appeal to youth. Participants further emphasized ongoing advocacy—regular campaigns, repeated messaging, and interactive activities—to reinforce non-smoking norms. This theme depicts students' support for integrating education, research, and policy enforcement can empower students and support a sustained tobacco-free culture.

The final theme emphasized the importance of collective action and partnership development, including stakeholder collaboration, student-led advocacy, and targeted action against flavored products. Participants viewed collaboration between universities, students, and health authorities as critical for creating tobacco-free environments. Working with local health institutions was seen as essential for awareness efforts, cessation support, and advocating for stricter regulation of flavored tobacco. University-led seminars involving health professionals were viewed as credible and effective. Social media collaborations with public health organizations were highlighted as important for countering misinformation, aligning with evidence that influencer-driven campaigns can reduce youth tobacco uptake (56). Participants strongly supported banning flavored tobacco and enforcing smoke-free campus policies, echoing WHO recommendations identifying flavors as major drivers of youth initiation and calling for comprehensive bans (2). These views are consistent with tobacco-free policy support in Qatar (57). Overall, the findings illustrate that partnerships—through institutional collaboration, student leadership, and policy enforcement—are welcomed by students for sustaining a tobacco-free campus and protecting youth from nicotine addiction. Despite Qatar's significant progress in the last two decades in tobacco legislation, awareness, and reductions in cigarette smoking, emerging products, persistent shisha culture, and enforcement gaps remain challenges. Continued efforts focused on policy implementation, youth-centered strategies, and expanded cessation support are critical (58–60). Future research should incorporate broader community perspectives during WNTD initiatives, as public engagement is essential for strengthening advocacy. Studies across diverse cultural and geographic settings would further support the development of comprehensive strategies that enhance public participation and improve the impact of tobacco-control messaging. These perspectives will reinforce the importance of student engagement in enhancing the acceptance of tobacco control initiatives when institutions invite them to be part of the process rather than imposing the policies.

### Limitations

This study has several limitations. The first limitation is related to the fact that the data was collected from a single university, which may limit generalizability to other institutions or settings, although this university is the largest in Qatar. Second, participants were individuals who visited the WNTD event booths, potentially over-representing those already interested in health or tobacco-control issues. Third, the cross-sectional design captures perceptions at one point in time and cannot assess changes in attitudes or behaviors longitudinally. Fourth, the study did not directly measure norms toward e-cigarettes; however, references to vaping emerged organically when participants elaborated on the questions. Although e-cigarette use exists in Qatar, strict regulations limiting sales and access may explain the relatively infrequent mention of vaping. Future research should explicitly assess e-cigarette norms to provide a comprehensive understanding of emerging nicotine products and the behaviors surrounding their use. Fifth, the sample was predominantly female, largely from health-related colleges, and mostly non-smokers, which underrepresents males, students from non-health clusters, and smokers. This may have influenced the perspectives shared and limits the transferability of the findings. Future studies should attempt to better represent these groups to ensure more diverse perspectives. Finally, although the analysis was informed by a phenomenological perspective, the use of brief written survey responses limited the depth of experiential insight, and findings should be interpreted as reflecting perceived meanings rather than comprehensive lived experiences. Nevertheless, the number of responses partially compensated for the loss in-depth that interviews offer but future studies should use interview to determine whether the depth of interpretation can be expanded further.

## Conclusion

This study contributes context-specific insight into how university students in Qatar understand and prioritize tobacco control within higher education settings, particularly in relation to the WNTD 2025 theme, “Unmasking the Appeal.” By tapping into student voices, the findings illustrate how tobacco use is perceived as a multidimensional issue shaped by health, mental wellbeing, social norms, education, and policy environments. A key contribution of this study is its demonstration that students view universities not only as sites for awareness-raising, but as active agents capable of shaping norms, policies, and advocacy against flavored tobacco and nicotine products. Participants emphasized the value of student-led engagement, credible health communication, curriculum integration, and partnerships with public health institutions as mechanisms to counter industry-driven appeal and reduce susceptibility to nicotine addiction. The unique study setting, which involved attending an awareness event prior to WNTD before completing the survey, is a strength; this approach can be improved upon through follow-up studies that are more inclusive of smokers, males, students from non-health programs, and replication across multiple universities. Overall, the study extends the limited Gulf-region literature on student engagement in tobacco control and offers practical, student-informed directions for universities seeking to translate global tobacco control themes into sustainable, locally relevant action.

## Data Availability

The data analyzed in this study is subject to the following licenses/restrictions: due to the sensitive nature of the data, the data is not available for publication. Requests to access these datasets should be directed to Mohammed Al-Hamdani, email: malhamdani@qu.edu.qa.

## References

[1] Brown JL Rosen D Carmona MG Parra N Hurley M Cohen JE. Spinning a global web: tactics used by big tobacco to attract children at tobacco points-of-sale. Tob Control. (2023) 32:645–51. doi: 10.1136/tobaccocontrol-2021-057095 35641117 PMC10447380

[2] World Health Organization (WHO). World No Tobacco Day 2025 - Unmasking the Appeal: Exposing Industry Tactics on Tobacco and Nicotine Products (2025). Available online at: https://www.who.int/news-room/events/detail/2025/05/31/default-calendar/world-no-tobacco-day-2025—unmasking-the-appeal (Retrieved August 20, 2025).

[3] Rutgers University Public Health Experts. Unmasking Big Tobacco's youth marketing playbook. Rutgers News (2025, May 27). Available online at: https://www.rutgers.edu/news/world-no-tobacco-day-2025-unmasking-big-tobaccos-youth-marketing-playbook (Retrieved August 20, 2025).

[4] Al-Hamdani M Manly E. Harm reduction in tobacco control: where do we draw the line? J. Public Health Policy. (2022) 43:149–54. doi: 10.1057/s41271-021-00327-5 34997211

[5] Al-hamdani M. A short note on e-cigarette issues: harm reduction, re-normalization, and big tobacco. J. Public Health Policy. (2014) 35:132–4. doi: 10.1057/jphp.2013.42 24107789

[6] Davidson M Al-Hamdani M. An examination of the social perceptions and vaping preferences of young electronic nicotine delivery system users. Front Public Health. (2023) 11:1150368. doi: 10.3389/fpubh.2023.1150368 37151590 PMC10162018

[7] Abid S Aggarwal A Duarte F Sethi S Iyengar S Zarich S. Nicotine pouches and youth: emerging patterns and potential cardiovascular risks. Front Public Health. (2025) 13:1632313. doi: 10.3389/fpubh.2025.1632313 41358246 PMC12675456

[8] Gravely S Cummings KM Hammond D Lindblom E Smith DM Martin N . The association of e-cigarette flavors with satisfaction, enjoyment, and trying to quit or stay abstinent from smoking among regular adult vapers from Canada and the United States: findings from the 2018 ITC four country smoking and vaping survey. Nicotine Tob Res. (2020) 22:1831–41. doi: 10.1093/ntr/ntaa095 32449933 PMC7542635

[9] Mosimann AF Güttinger EM Tal K Schoeni A Baggio S Sambiagio N . E-liquid flavors and nicotine concentration choices over 6 months after a smoking cessation attempt with ENDS: secondary analyses of a randomized controlled trial. Tobacco Prev Cessat. (2025) 11:4. doi: 10.18332/tpc/196136 PMC11734314 39816168

[10] Al-Hamdani M Manly E. Smoking cessation or initiation: the paradox of vaping. Preventive medicine reports. (2021) 22:101363. doi: 10.1016/j.pmedr.2021.101363 33868902 PMC8044675

[11] Hoffman AC Salgado RV Dresler C Faller RW Bartlett C. Flavour preferences in youth versus adults: a review. Tob Control. (2016) 25:ii32–9. doi: 10.1136/tobaccocontrol-2016-053192 27633764 PMC5127592

[12] Rose SW Johnson AL Glasser AM Villanti AC Ambrose BK Conway K . Flavour types used by youth and adult tobacco users in wave 2 of the population assessment of tobacco and health (PATH) study 2014–2015. Tob Control. (2020) 29:432–46. doi: 10.1136/tobaccocontrol-2018-054852 31542778 PMC7462091

[13] Talhout R Leventhal AM. Coolants, organic acids, flavourings and other additives that facilitate inhalation of tobacco and nicotine products: implications for regulation. Tobacco Control. (2026) 35:110–6. doi: 10.1136/tc-2024-058738 39256038 PMC12911559

[14] Ford A MacKintosh AM Bauld L Moodie C Hastings G. Adolescents' responses to the promotion and flavouring of e-cigarettes. Int J Public Health. (2016) 61:215–24. doi: 10.1007/s00038-015-0769-5 26650455 PMC4819499

[15] Wang L Chen J Ho SY Leung LT Wang MP Lam TH. Exposure to e-cigarette advertising, attitudes, and use susceptibility in adolescents who had never used e-cigarettes or cigarettes. BMC Public Health. (2020) 20:1349. doi: 10.1186/s12889-020-09422-w 32887586 PMC7650221

[16] Saffer H Ozdogan S Grossman M Dench D Dave D. Comprehensive e-cigarette flavor bans and tobacco use among youth, young adults, and adults. Health Econ. (2025). doi: 10.3386/w32534 PMC12645907 40898822

[17] Ambrose BK Day HR Rostron B Conway KP Borek N Hyland A . Flavored tobacco product use among US youth aged 12-17 years, 2013-2014. JAMA. (2015) 314:1871. doi: 10.1001/jama.2015.13802 26502219 PMC6467270

[18] Vogel EA Ramo DE Rubinstein ML Delucchi KL Darrow SM Costello C . Effects of social media on adolescents' willingness and intention to use e-cigarettes: an experimental investigation. Nicotine Tob Res. (2021) 23:694–701. doi: 10.1093/ntr/ntaa003 31912147 PMC7976937

[19] Tobacco Tactics. Tobacco Industry Targeting Young People (2025, August 14). Available online at: https://www.tobaccotactics.org/article/tobacco-industry-targeting-young-people/ (Retrieved August 31, 2025).

[20] Reitsma MB Kendrick PJ Ababneh E Abbafati C Abbasi-Kangevari M Abdoli A . Spatial, temporal, and demographic patterns in prevalence of smoking tobacco use and attributable disease burden in 204 countries and territories, 1990–2019: a systematic analysis from the Global Burden of Disease Study 2019. Lancet. (2021) 397:2337–60. 34051883 10.1016/S0140-6736(21)01169-7 PMC8223261

[21] Seaman Jones EL Ali FR Kreslake JM Gentzke AS Schillo BA Marynak K. Stronger flavor policies, better outcomes for young people: comparing youth and young adult tobacco use behaviors in areas with and without flavored tobacco sales restrictions, by strength of policy, 2022. JNCI Monographs. (2025) 2025:235–42. doi: 10.1093/jncimonographs/lgaf019 40795909

[22] Harrell MB Weaver SR Loukas A Creamer M Marti CN Jackson CD . Flavored e-cigarette use: characterizing youth, young adult, and adult users. Preventive medicine reports. (2016) 5:33–40. doi: 10.1016/j.pmedr.2016.11.001 27896041 PMC5121224

[23] Berry KM Fetterman JL Benjamin EJ Bhatnagar A Barrington-Trimis JL Leventhal AM . Association of electronic cigarette use with subsequent initiation of tobacco cigarettes in US youths. JAMA network open. (2019) 2:e187794. doi: 10.1001/jamanetworkopen.2018.7794 30707232 PMC6484602

[24] Baenziger ON Ford L Yazidjoglou A Joshy G Banks E. E-cigarette use and combustible tobacco cigarette smoking uptake among non-smokers, including relapse in former smokers: umbrella review, systematic review and meta-analysis. BMJ Open. (2021) 11:e045603. doi: 10.1136/bmjopen-2020-045603 PMC8011717 33785493

[25] Dai H Leventhal AM. Association of electronic cigarette vaping and subsequent smoking relapse among former smokers. Drug Alcohol Depend. (2019) 199:10–7. doi: 10.1016/j.drugalcdep.2019.01.043 30978519 PMC6743978

[26] Al-Hamdani M Davidson M Bird D Hopkins DB Smith S. Learning from their experiences: strategies used by youth and young adult ex-vapers. J. Substance Use Add Treat. (2023) 149:209038. doi: 10.1016/j.josat.2023.209038 37061190

[27] Monshi SS Wu J Collins BN Ibrahim JK. Youth susceptibility to tobacco use in the Gulf cooperation council countries, 2001–2018. Prev Med Reports. (2022) 26:101711. doi: 10.1016/j.pmedr.2022.101711 PMC8819131 35145839

[28] Knowledge Action Change. Country Profile: Qatar (2025). Available online at: https://gsthr.org/countries/profile/qat/ (Retrieved January 17, 2026).

[29] Al-hamdani M. Plain packaging policy: preventing industry innovations. Canadian J Public Health. 108:e98-e100. doi: 10.17269/CJPH.108.5828 PMC6975215 28425907

[30] Moodie C Angus K Stead M. Consumer response to standardized tobacco packaging in the United Kingdom: a synthesis of evidence from two systematic reviews. Risk Manag Healthc Policy. (2021) 1465–80. doi: 10.2147/RMHP.S272259 33883953 PMC8053612

[31] Al-Hamdani M Hopkins DB. E-cigarettes in the Middle East: the known, unknown, and what needs to be known next. Prev Med Reports. (2023) 31:102089. doi: 10.1016/j.pmedr.2022.102089 PMC9747636 36530454

[32] Moodie C Hoek J Hammond D Gallopel-Morvan K Sendoya D Rosen L . Plain tobacco packaging: progress, challenges, learning and opportunities. Tob Control. (2022) 31:263–71. doi: 10.1136/tobaccocontrol-2021-056559 35241599

[33] McDaniel PA Smith EA Malone RE. The tobacco endgame: a qualitative review and synthesis. Tob Control. (2016) 25:594–604. doi: 10.1136/tobaccocontrol-2015-052356 26320149 PMC5036259

[34] Knowledge Action Change. Qatar: Cigarettes (2023). Available online at: https://gsthr.org/countries/profile/qat/cigarettes/?utm_source=chatgpt.com (Retrieved August 6, 2025).

[35] ATLAS.ti Scientific Software Development GmbH. ATLAS.ti Mac (version 23.2.1) [Qualitative Data Analysis Software] (2023). Available online at: https://atlasti.com (Retrieved August 6, 2025).

[36] Braun V Clarke V. Using thematic analysis in psychology. Qual Res Psychol. (2006) 3:77–101. doi: 10.1191/1478088706qp063oa

[37] Siddiqi K Dogar OF Siddiqi N. Smoking cessation in long-term conditions: is there “an opportunity in every difficulty”? Int J Population Res. (2013) 107:251048. doi: 10.1155/2013/251048

[38] Frey P Waters DD DeMicco DA Breazna A Samuels L Pipe A . Impact of smoking on cardiovascular events in patients with coronary disease receiving contemporary medical therapy (from the Treating to New Targets [TNT] and the incremental decrease in end points through aggressive lipid lowering [IDEAL] trials). Am J Cardiol. (2011) 107:145−50. doi: 10.1016/j.amjcard.2010.09.006 21129718

[39] Abdulhay N Wiener RC Wen S Gibbs BB Bhandari R. Mental health outcomes associated with electronic cigarette use, combustible tobacco use, and dual use among US adolescents: insights from the national youth tobacco survey. PLOS Mental Health. (2025) 2:e0000370. doi: 10.1371/journal.pmen.0000370 41661972 PMC12798231

[40] Erath TG Chen FF DeSarno M Devine D Leventhal AM Bickel WK . Cumulative psychosocial and health disparities in US adolescent cigarette smoking, 2002-2019. JNCI: J Nat Cancer Inst. (2025) 117:665–72. doi: 10.1093/jnci/djae286 39535862 PMC11972670

[41] VanFrank B., Williams, T. R., Alcantara, I. C., Hertz, M., Al-Shawaf, M., Meyers, C. (2025). E-cigarette use and symptoms of depression and anxiety among U.S. middle and high school students. Prev Chronic Dis. 22:250186. doi: 10.5888/pcd22.250186 PMC12360061 40815093

[42] Lawless MH Harrison KA Grandits GA Eberly LE Allen SS. Perceived stress and smoking-related behaviors and symptomatology in male and female smokers. Addict Behav. (2015) 51:80–3. doi: 10.1016/j.addbeh.2015.07.011 26240941 PMC4558262

[43] Taylor GMJ Baker AL Fox N Kessler DS Aveyard P Munafò MR. Addressing concerns about smoking cessation and mental health: a theoretical review and practical guide. BJPsych Advances. (2020) 27:85–95. doi: 10.1192/bja.2020.52 34513007 PMC7611646

[44] Do EK Fallavollita WL Bonat B Fugate-Laus K Rossi BC Fuemmeler BF. Student attitudes toward tobacco use and tobacco policies on college campuses. J Community Health. (2020) 45:751–60. doi: 10.1007/s10900-020-00790-3 31925604 PMC8058647

[45] Liu J Zhao S Chen X Falk E Albarracín D. The influence of peer behavior as a function of social and cultural closeness: a meta-analysis of normative influence on adolescent smoking initiation and continuation. Psychol Bull. (2017) 143:1082–115. doi: 10.1037/bul0000113 28771020 PMC5789806

[46] Chaaya M Farran D Saab D Al-Hindi M Romani M Khairallah M . Influence of a university tobacco-free policy on the attitudes, perceptions of compliance, and policy benefit among the university students: a pre-post investigation. Int J Public Health. (2021) 66:614602. doi: 10.3389/ijph.2021.614602 34744578 PMC8565293

[47] Lakon CM Zheng Y Pechmann C. Social network tie functions of social support and social influence and adult smoking abstinence. PLoS ONE. (2024) 19:e0296458. doi: 10.1371/journal.pone.0296458 38452042 PMC10919666

[48] Issa F Abdulla M Abouzoor R Abdallah AM Al-Hamdani M. Second-hand smoke exposure level in the household increases risk of chest pain and wheezing: evidence from Qatar biobank. BMC Public Health. (2025) 25:2698. doi: 10.1186/s12889-025-23927-2 40775691 PMC12330130

[49] Gasparotto J., Berlowitz, D. (2025). Editorial: world no-tobacco day: an ethnopharmacological perspective. Front *Pharmacol*. 16:1538085. doi: 10.3389/fphar.2025.1538085 PMC11862690 40012628

[50] MacMonegle A Bennett M Speer JL O'Brien EK Pitzer L Jaarsma A . Evaluating the real cost digital and social media campaign: longitudinal effects on e-cigarette beliefs. Nicotine Tob Res. (2024) 26:S19–26. doi: 10.1093/ntr/ntad185 38366338

[51] Allom V Jongenelis M Slevin T Keightley S Phillips F Beasley S . Comparing the cost-effectiveness of campaigns delivered via various combinations of television and online media. Front Public Health. (2018) 6:83. doi: 10.3389/fpubh.2018.00083 29629366 PMC5876296

[52] Daramola I Ezeagu CN Aderinto N Olatunji G Kokori E. Short videos, big impact: WHO and TikTok join forces. Discover Public Health. (2025) 22:1–3. doi: 10.1186/s12982-025-00777-2

[53] Li J Li C Liu M. The role of health education, policies, and services on college students' health behavior. Am J Health Behav. (2022) 46:618–26. doi: 10.5993/AJHB.46.6.4 36721280

[54] Maziak W Taleb ZB Bahelah R Islam F Jaber R Auf R . The global epidemiology of waterpipe smoking. Tob Control. (2015) 24:i3–i12. doi: 10.1136/tobaccocontrol-2014-051903 25298368 PMC4345835

[55] Shi Y Qin D Wang H Sun L Gu K. Effectiveness of tobacco control policy interventions on tobacco use behaviours and health outcomes based on ITS research methodology: a scoping review. BMJ Open. (2025) 15:e094148. doi: 10.1136/bmjopen-2024-094148 PMC12587966 41248326

[56] Lin S Koch JR Barnes AJ Hayes RB Xue H. Assessing the effects of social media based anti-tobacco campaigns on tobacco use among youth in Virginia: an agent-based simulation approach. Eur J Public Health. (2025) 35:924–9. doi: 10.1093/eurpub/ckaf085 40650327 PMC12529265

[57] Al-Naimi A Elsayed K Alharoon M Al-Obaidli F Almuraikhi H Osman A . Tobacco endgame policies: an analysis of preferred strategies and support levels in a sample from Qatar. Front Public Health. (2025) 13:1515633. doi: 10.3389/fpubh.2025.1515633 40552217 PMC12183201

[58] Al-Kuwari MG Al-Khenji AA Al-Baker WA Bala MO. Tobacco control in Qatar from 2002 to 2022: 20 years of progress and challenges. J Public health Res. (2024) 13. doi: 10.1177/22799036241229341 PMC10846134 38322020

[59] Ranabhat CL Kim CB Park MB Jakovljevic M. Situation, impacts, and future challenges of tobacco control policies for youth: an explorative systematic policy review. Front Pharmacol. (2019) 10:981. doi: 10.3389/fphar.2019.00981 31551784 PMC6745506

[60] Park E Zhou Y Chen C Chacko T Mahoney M Chang YP. Systematic review: interventions to quit tobacco products for young adults. BMC Public Health. (2023) 23:1233. doi: 10.1186/s12889-023-15900-8 37365562 PMC10294369

